# School, Supervision and Adolescent-Sensitive Clinic Care: Combination Social Protection and Reduced Unprotected Sex Among HIV-Positive Adolescents in South Africa

**DOI:** 10.1007/s10461-016-1539-y

**Published:** 2016-09-08

**Authors:** Elona Toska, Lucie D. Cluver, Mark E. Boyes, Maya Isaacsohn, Rebecca Hodes, Lorraine Sherr

**Affiliations:** 1 Centre for Evidence-Based Intervention, Department of Social Policy & Intervention, University of Oxford Barnett House, 32 Wellington Square, Oxford, OX1 2ER UK; 2 AIDS and Society Research Unit, Centre for Social Science Research, University of Cape Town, 4.26 Leslie Building, Private Bag Rondebosch, Cape Town, Western Cape 7701 South Africa; 3 Department of Psychiatry and Mental Health, Groote Schuur Hospital, University of Cape Town, J-Block, Observatory, Cape Town, 7925 South Africa; 4 Health Psychology and Behavioural Medicine Research Group, School of Psychology and Speech Pathology, Curtin University, Perth, WA Australia; 5 Tulane University School of Medicine, 1430 Tulane Ave, New Orleans, LA 70112 USA; 6 University College London, London, UK

**Keywords:** HIV-positive adolescents, Social protection, Unprotected sex, Secondary prevention, South Africa

## Abstract

Social protection can reduce HIV-risk behavior in general adolescent populations, but evidence among HIV-positive adolescents is limited. This study quantitatively tests whether social protection is associated with reduced unprotected sex among 1060 ART-eligible adolescents from 53 government facilities in South Africa. Potential social protection included nine ‘cash/cash-in-kind’ and ‘care’ provisions. Analyses tested interactive/additive effects using logistic regressions and marginal effects models, controlling for covariates. 18 % of all HIV-positive adolescents and 28 % of girls reported unprotected sex. Lower rates of unprotected sex were associated with access to school (OR 0.52 95 % CI 0.33–0.82 *p* = 0.005), parental supervision (OR 0.54 95 % CI 0.33–0.90 *p* = 0.019), and adolescent-sensitive clinic care (OR 0.43 95 % CI 0.25–0.73 *p* = 0.002). Gender moderated the effect of adolescent-sensitive clinic care. Combination social protection had additive effects amongst girls: without any provisions 49 % reported unprotected sex; with 1–2 provisions 13–38 %; and with all provisions 9 %. Combination social protection has the potential to promote safer sex among HIV-positive adolescents, particularly girls.

## Resumen

La protección social puede reducir los comportamientos de riesgo asociados al VIH en los adolescentes en general, siendo los datos limitados en cuanto a adolescentes VIH-positivo se refiere. Este estudio se evalúa cuantitativamente si la protección social está asociada con la reducción de relaciones sexuales sin protección en una muestra de 1060 adolescentes elegibles para el tratamiento antirretroviral, en 53 instalaciones gubernamentales en Sudáfrica. En este estudio la protección social se midió usando nueve tipos de medidas de protección incluyendo efectivo, pago en especie y servicios de atención a la salud. Los efectos interactivos y aditivos de estas medidas se analizaron usando regresiones logísticas y modelos de efectos marginales, controlando por covariables. El 18 % de todos los adolescentes VIH positivo y el 28 % de las chicas adolescentes declararon haber tenido relaciones sexuales sin protección. Menores tasas de relaciones sexuales sin protección estuvieron asociadas con el acceso a la escuela (OR 0.52 95 % CI 0.33–0.82 *p* = 0.005), la supervisión parental (OR 0.54 95 % CI 0.33–0.90 *p* = 0.019), y la atención clínica adecuada a las necesidades de los adolescentes (OR 0.43 95 % CI 0.25–0.73 *p* = 0.002). El género moderó el efecto de la atención clínica adecuada a las necesidades de los adolescentes. La combinación de medidas de protección social tuvo efectos en las chicas adolescentes: sin ninguna medida de atención, el 49 % de las chicas declaro haber mantenido relaciones sexuales sin protección, mientras que con una o dos medidas el 13–38 %, y con todas las medidas solo el 9 %. La combinación de medidas de protección social tiene el potencial de promover relaciones sexuales seguras en los adolescentes VIH positivo, en particular en las chicas adolescentes.

## Introduction

There are an estimated 1.3–2.2 million HIV-positive adolescents in Sub-Saharan Africa, both vertically and horizontally infected [1]. Studies have documented high rates of unprotected sex reported by HIV-positive adolescents even after HIV infection (27–90 %) [2–5]. While rates of unprotected sex among HIV-positive adolescents are comparable to those among the general adolescent population [2], HIV-positive adolescents are a key population for reducing onwards HIV transmission to sexual partners and children. In addition, HIV-positive adolescents experience a range of vulnerabilities that are likely to reduce the efficacy of HIV prevention programmes aimed at general populations, including cognitive and mental health issues [6, 7], family-related challenges [8, 9] and material deprivation [10, 11].

Adolescent girls and young women bear a disproportionate burden of the epidemic: three-quarters of all new HIV infections in Africa are among adolescent girls, and 80 % of all HIV-positive adolescent girls live in Sub-Saharan Africa [12, 13]. While notable research and resources are focused on supporting adolescent girls and young women to remain HIV-negative, there is a dearth of research and programming for HIV-positive girls. HIV-positive adolescent girls face multiple potential risks: low rates of condom and contraceptive use, greater rates of unwanted pregnancies and related health complications, as well as lower enrollment, adherence to, and retention in prevention-of-mother-to-child transmission programmes, and, consequently, increased risk of transmitting HIV to their partners and children [14–18].

Increasingly, social protection provisions are showing potential to reduce the negative impacts of structural deprivations faced by adolescents in high-prevalence contexts, and to improve their long-term health outcomes [19]. Although traditionally defined as a set of economic measures such as welfare payments or social cash transfers, recent conceptualisations of social protection recognise that it may take one of multiple forms [19–21]: ‘cash/cash-in-kind’ provisions to address economic barriers to food security, school access and health services, or psychosocial ‘care’ provisions such as support groups, supportive parenting or community services [22]. Most evidence to date has focused on impacts of social cash transfers in addressing structural vulnerabilities to HIV-infection among adolescents in Sub-Saharan Africa [13]. But recent studies suggest that combinations of ‘cash/cash-in-kind’ and ‘care’ social protection provisions may have greater potential for reducing HIV risk-behaviour than single interventions [23, 24]. Two studies from South Africa and Kenya suggest that social protection may function differently for boys and girls [23, 25]. A longitudinal study of n = 2668 South African adolescents found that different combinations of ‘cash’ and ‘care’ social protection were associated with reductions in sexual risk-taking among adolescent girls compared to adolescent boys [23]. The evaluation of the Kenya cash transfer programme for orphans and vulnerable children showed overall reductions in sexual debut with greater impact among girls compared to boys [25]. A recent review in Eastern and Southern Africa reported an increasing evidence base on how social protection can reduce HIV infection among HIV-negative adolescents, but found no studies that investigate the role of social protection in preventing onwards HIV-transmission among HIV-positive adolescents [21]. There is a need for evidence on whether social protection provisions alone or in combination can reduce HIV-risk behavior for HIV-positive adolescents, and to understand potential gender differences.

To date, only a few programmes have tested any interventions to improve sexual and reproductive health among HIV-positive adolescents in Sub-Saharan Africa. A small-scale randomised trial of a behavioural intervention among 14–21 year old HIV-positive youth in Uganda reported that intervention youth (n = 50) increased consistent condom use and reduced number of sexual partners significantly compared to controls (n = 50) [26]. Three studies suggest that ‘care’ interventions of support groups may be helpful in reducing risk behaviors amongst HIV-positive adolescents [27–29]. A pre-post test pilot study of structured support group sessions for HIV-positive adolescents (n = 65) in South Africa found improvements in self-reported condom use [27]. A qualitative study (n = 13) in the Democratic Republic of Congo, consisting of a 6-session group-based healthy living intervention reported better communication with sexual partners [29]. However, no large-scale or quantitative research has examined impacts of either ‘cash/cash-in-kind’ or ‘care’ social protection provisions, alone or in combination, on the sexual practices of HIV-positive adolescents. Combination social protection may have cumulative effects, that is beneficiaries of two or more provisions may do better than those receiving each provision alone. These effects may be multiplicative or additive [23].

This study aims to address this essential research gap. It uses the world’s largest community-traced sample of HIV-positive adolescents to investigate whether different types of social protection provisions: ‘cash/cash-in-kind’ or ‘care’, are associated with lower rates of unprotected sex. Based on a review of literature on social protection for HIV prevention [21], the following nine social protection provisions were tested: ‘cash/cash-in-kind’: social cash transfers, past-week food security, free school access (no fees and school materials), school feeding, and clothing, and psychosocial ‘care’ provisions: positive parenting, strong parental supervision, support groups, adolescent-sensitive care at clinics (respectful treatment by sexual health service providers). It tests (1) associations of each social protection provisions with unprotected sex, (2) the effects of gender on social protection provisions significantly associated with unprotected sex, (3) potential interactive effects of significant social protection provisions, and (4) potential additive effects of combination social protection provisions.

## Methods

### Participants and Procedures

1060 HIV-positive adolescents (10–19 year olds) were recruited from a health district in the Eastern Cape province, South Africa. This was selected as a resource-limited setting with high HIV-prevalence rates [30]. The study was designed in collaboration with South African Departments of Health and Basic Education, UNICEF, PEPFAR-USAID, Pediatric AIDS Treatment for Africa (PATA) and local NGOs. Ethical approval for this study was provided by Research Ethics Committees at the Universities of Oxford (SSD/CUREC2/12-21) and Cape Town (CSSR 2013/4), Eastern Cape Departments of Health and Basic Education, and ethical review boards of participating hospitals.

The study aimed to include all 10–19 year old adolescents within the health district who were eligible to initiate ART. First, all healthcare facilities providing ART were visited (n = 83): all facilities who reported more than five ART-eligible adolescents were included in the study (n = 39). As the study progressed, the South African Department of Health implemented a primary healthcare reengineering programme, as a result of which the adolescents receiving care in the initial 39 facilities were transferred to a total of 53 healthcare facilities including hospitals, community healthcare centres, and primary healthcare clinics. All 53 facilities were then included in the study.

Adolescents were recruited at clinics where they were receiving antiretroviral treatment and care, or traced into their home communities for those not reachable at the clinics. All caregivers and adolescents participating in the study gave written informed consent prior to interviews, which took place in the language of their choice and lasted an average of 90 min. Of all study-eligible adolescents, n = 1060 (90.1 %) were interviewed, 4.1 % refused participation (either adolescent or caregiver), 0.9 % were excluded due to severe cognitive disability, 1.2 % were excluded due to living in very unsafe areas, and 3.7 % were untraceable. Participants who asked for help or disclosed abuse, neglect, defaulting from antiretroviral treatment or clinic care, severe hunger, or risk of significant harm were immediately assisted and linked to existing services (n = 66, 6.2 %). Due to high HIV-stigma rates, the study was presented in participating communities as a general study on adolescent access to health and social services. In order not to draw attention to HIV-affected families, when participants were traced and interviewed in communities, an additional n = 467 cohabitating or neighbouring age-peers were interviewed using a non HIV-specific version of the questionnaire (not included in this analysis).

Quantitative and qualitative research were combined iteratively during the study: qualitative research guided the design and content of the quantitative data collection tools and processes, preliminary quantitative analysis provided themes to be further explored by qualitative research, and these in-depth explorations shaped quantitative analyses. Quantitative questionnaires used standardised scales and validated measures when available. Tools were translated into Xhosa and back-translated for improved conceptual validity [31], then piloted with 25 HIV-positive adolescents from rural and urban sites in the health district. Questionnaires included graphics, interactive games and vignettes to introduce questions around sensitive topics. Interviews were administered by trained research assistants or via tablet-assisted self-interviewing, based on the participants’ literacy levels.

### Measures

Unprotected sex at last sexual intercourse was measured as no condom use at most recent sexual encounter. It was dichotomised as: ‘1 = unprotected sex’ and ‘0 = abstinence or protected sex’. Adolescents were coded as STI symptomatic if they reported having at least one of the following four STI symptoms: genital sores/warts, burning whilst urinating, genital itching/redness, or anal itching/soreness/bleeding, in the last 6 months, following WHO guidelines for syndromatic diagnosis of STIs [32]. Adolescent pregnancy among girls was defined as ever having been pregnant before or during data collection, measured using an item from the National Survey of HIV and Risk Behaviour Amongst Young South Africans [33].

Socio-demographic characteristics (age, gender, home language, housing situation, urban/rural location) were measured using items from South Africa’s Census [34]. Housing was coded as 1 = informal if the adolescent lived in a hut, rondavel (traditional home), or a shack, and 0 = formal if they lived in a brick/concrete house or apartment. Orphanhood status was coded as death of either mother or father or both [35].

#### HIV-Related Factors

Mode of infection was assessed following similar studies and modelling from Southern Africa [36, 37]: adolescents were coded as vertically-infected if they had started ART prior to age 12 or if they had been on treatment for more than 5 years, based on the year of widely available ART access in the study area. Adolescent’s knowledge of their own HIV-positive status was determined through a stepwise process: initially healthcare providers’ report, followed by confirmation by caregiver during the consent process. Additional checks on adolescent knowledge of own HIV-status were conducted using a screening on recent health and medication-taking histories to avoid unintentional disclosure. Adolescents who did not know their own HIV-positive status responded to a questionnaire on ‘illness’ and ‘medication’ instead of ‘HIV’ and ‘antiretrovirals’, respectively. Most recent viral loads were extracted from patient records for a random sub-sample (n = 266, 25 %). Participants with viral load counts >1000 copies/ml were coded as reporting virological failure using WHO standards [38].

#### Social Protection Provisions

‘Cash/cash-in-kind’ provisions of social protection included the following: Social cash transfers referred to participants’ household receiving at least one of South Africa’s five social welfare grants: child support grant, foster child grant, pension, disability or care dependency. Past-week food security, defined as at least two meals daily for the past week, was measured through items from the National Food Consumption Survey [39]. Access to school was defined as access to free schooling or ability to afford school fees, uniform and equipment. School feeding referred to receiving at least one free meal at school daily. Sufficient clothing was measured using an item from the South African Social Attitudes Survey [40]. Psychosocial ‘care’ provisions included: Positive parenting—including items on praise and positive reinforcement from caregiver—and good parental supervision—including monitoring of adolescent social activities and home rule-setting—measured using two sub-scales of the Alabama Parenting Questionnaire [41]. Attending an HIV-support group was measured as past-month attendance at either a youth-focused or general HIV-support group. Adolescent-sensitive care at clinics was measured through two items asking adolescents about their experience obtaining contraception at the clinic: whether they felt disrespected or were scolded. These items were developed based on extensive qualitative research and consultations with HIV-positive adolescents in the study’s teen advisory group [15].

### Data Analysis

Data analysis consisted of five steps: first, the included sample (90.1 %) was compared to the rest of the eligible sample across available key demographics (age, gender and residential location) to check for any differences. Descriptive statistics of socio-demographic characteristics, access to each social protection provision, and rates of unprotected sex were calculated for the full included sample and by gender. Covariates and social protection provisions were excluded from further analysis if sub-group sizes were too small for reliable analysis (cut-off n < 100 in the full sample, n < 50 per gender). To check the extent of risk for onwards HIV-transmission, we tested whether unprotected sex was associated with virological failure, a marker of high HIV-transmission risk through unprotected sex [42].

Second, validation checks for self-reported unprotected sex were conducted by testing associations between a) unprotected sex and STI symptomology (full sample) and b) unprotected sex and pregnancy (females only). These used multivariate logistic regression models controlling for all potential covariates.

Third, we tested potential associations of unprotected sex and seven social protection provisions: three ‘cash-in-kind’ and four ‘care’, using a multivariate logistic regression model, controlling for covariates. Covariates entered included: adolescent age, gender, language, housing type, residential location, maternal and paternal orphanhood, living with biological caregiver, mode of infection, and knowledge of own HIV-positive status.

Fourth, we tested whether gender acted as a moderator for each social protection provision. Moderator analyses were conducted using logistic regression models with two-way interaction terms of gender and each social protection provisions entered in separate models, controlling for covariates found significant in the above step. Subsequently, based on existing literature suggesting different social protection provisions may work for adolescent boys and girls, and because a moderator effect was found, multivariate logistic regressions were run separately for HIV-positive girls and boys.

Fifth, effects of combinations of social protection provisions on unprotected sex were tested for the full and then gender-disaggregated samples. To check for potential interaction effects, all significant social protection variables, covariates and interaction terms from stage 3 above (p < .05) were added in a stepwise multivariate logistic regression model, following processes applied by similar studies [23]. Step 1—all covariates significant from the model in stage 3, step 2—all significant social protection variables, step 3—all two-way interaction terms of significant social protection variables, step 4—all three-way and higher order interaction terms of significant social protection variables. Subsequently, marginal effect analysis in STATA tested potential additive effects of significant social protection provisions by computing predicted probabilities of unprotected sex under each potential combination of significant social protection provisions, with all significant covariates held at mean values.

## Results

### Socio-Demographic and HIV-Related Factors (Table 1)

Over half the sample was female (55 %) with average age 13.8 (SD = 2.8). 19 % lived in informal housing. 22 % lived in rural areas. Almost all participants spoke Xhosa at home (97 %) and just under half lived with a biological caregiver (45 %). 44 % were maternal orphans, 30 % paternal orphans, and 15.4 % had lost both parents. 67 % were vertically-infected and 75 % knew their own HIV-positive status. Due to small sub-sample sizes of non-Xhosa speakers (<50 for each gender), home language was excluded from further analyses. There were no significant differences between the included (n = 1060) and excluded eligible participants (n = 116), when compared across age, gender and residential location.

**Table 1:** Socio-demographic characteristics of the sample by gender

Factor grouping	Factor	Excluded eligible sample n = 166^a^ N (%)^b^	Included eligible sample n = 1060^a^ N (%)	Total sample n = 1060	
				Female n = 584 (55.1 %)	Male n = 476 (44.9 %)
Age	Years [mean (SD)]	14.8 (2.91)	13.8 (2.8)	14.3 (3.0)	13.3 (2.5)
	10–14		659 (62.2)	324 (55.5)	335 (70.4)
	15–19		401 (37.8)	260 (44.5)	141 (29.6)
Gender	Female	66 (56.9)	584 (55.1)	584 (100)	n/a
Language	Xhosa		1028 (97.0)	572 (97.9)	456 (95.8)
Housing	Formal		861 (81.3)	469 (80.3)	392 (82.5)
	Informal		198 (18.7)	115 (19.7)	83 (17.5)
Residence	Urban	140 (77.6)	828 (78.4)	451 (77.5)	377 (79.5)
	Rural	26 (22.4)	228 (21.6)	131 (22.5)	97 (20.5)
Family and caregiver characteristics	Maternal orphan		464 (43.8)	250 (42.8)	214 (45.0)
	Paternal orphan		320 (30.2)	183 (31.3)	137 (28.8)
	Living with biological caregiver		476 (44.9)	275 (47.1)	201 (42.2)
HIV-related factors	Vertical infection		708 (66.8)	348 (59.6)	360 (75.6)
	Horizontal infection		352 (33.2)	236 (40.4)	116 (24.4)
	Knows HIV-positive status		794 (74.9)	442 (75.7)	352 (73.9)

^a^Statistical tests comparing the excluded and included eligible participants were non significant

^b^N (%) reported unless noted otherwise

### Sexual Outcomes: (Table 2)

18 % of HIV-positive adolescents reported having unprotected sex at last intercourse, with girls reporting significantly higher rates than boys (28 % vs. 4 %, OR 8.46, 95 % CI 5.27–13.58 *p* ≤ .001). 32 % of HIV-positive girls were STI symptomatic compared to 27 % of boys (OR 1.23 95 % CI 0.99–1.69 *p* = 0.059), with 13 % of all HIV-positive girls reporting past or current pregnancy.

**Table 2:** Outcome measures and access to social protection provisions by gender

Factor grouping	Factor	Female n = 584 (55.1 %)	Male n = 476 (44.9 %)	Total n = 1060 (100 %)
Outcome	Unprotected sex at last intercourse	164 (28.1)	21 (4.4)	185 (17.5)
	STI symptomatic	187 (32.0)	127 (26.7)	314 (29.6)
	Pregnant (current or ever)	78 (13.4)	n/a	n/a
	Virological failure^a^	33 (24.8)	35 (26.3)	68 (25.6)
Economic ‘cash/cash-in-kind’ social protection provisions	Social cash transfers	553 (94.7)	450 (94.7)	1003 (94.7)
	Food security	431 (73.8)	389 (81.7)	820 (77.4)
	Access to school	355 (60.8)	344 (72.3)	699 (65.9)
	School feeding	538 (92.1)	448 (94.1)	986 (93.0)
	Clothing	393 (67.3)	318 (66.8)	711 (67.1)
Psychosocial ‘care’ social protection provisions	Positive parenting	298 (51.0)	233 (49.1)	531 (50.1)
	Good parental supervision	227 (38.9)	206 (43.4)	433 (40.9)
	HIV support group	76 (13.0)	65 (13.7)	141 (13.3)
	Adolescent-sensitive clinic care	487 (83.4)	437 (91.8)	924 (87.2)

Virological failure defined as >1000 copies/ml

^a^Sample size for viral load data n = 266, n = 133 girls (50 %) and n = 133 boys (50 %)

### Transmission Risk

Unprotected sex was strongly associated with virological failure in the sub-sample for whom viral load data was available (n = 266, OR 2.57 95 % CI 1.01–6.53 *p* = 0.048), suggesting that a sub-group of HIV-positive adolescents who are not virally suppressed and engage in unprotected sex are at high risk for HIV-transmission to uninfected sexual partners and children. Gender-disaggregated analyses were not possible due to small sub-sample sizes.

### Access to Social Protection Provisions (Table 2)

‘Cash/cash-in-kind’: 95 % of adolescents reported that their household received at least one cash grant and 77 % had enough food to eat in the past week. 66 % had no economic barriers to access school, 93 % received regular school feeding, and 67 % had enough clothes to stay warm and dry. ‘Care’: 13 % attended any HIV support group, 41 % reported high parental supervision and 50 % reported high positive parenting. HIV-positive adolescent boys reported higher rates of food security (*Χ* ^2^ (df) = 9.395 [1], *p* = 0.002), greater access to school (*Χ* ^2^ (df) = 15.393 [1], *p* ≤ 0.001), and more adolescent-sensitive SRH care at clinics (*Χ* ^2^ (df) = 16.610 [1], *p* ≤ 0.001) than girls. Due to the very small groups of adolescents not receiving social cash transfers and school feeding schemes (<100 in the full sample, <50 by gender), these provisions were excluded from further analyses.

### Validating Self-Reported Unprotected Sex (Table 3)

In multivariate logistic regression, self-reported unprotected sex was strongly associated with STI symptomology in the full sample (OR 1.54 95 % CI 1.00–2.38 *p* = 0.05) and with adolescent pregnancy among girls only (OR 5.72 95 % CI 2.51–13.03 *p* ≤ 0.001).

**Table 3:** Associations of unprotected sex with pregnancy and STI symptomology among HIV-positive adolescent

Factors	Model 1: HIV-positive adolescents girls (n = 584)		Model 2: HIV-positive adolescents (n = 1060)	
	OR (95 % CI)	p	OR (95 % CI)	p
Outcome: unprotected sex at last intercourse				
Age	1.607 (1.419–1.819)	≤.001	1.723 (1.554–1.910)	≤.001
Gender	Not entered in model		6.591 (3.884–11.155)	≤.001
Informal housing	.832 (.455–1.522)	.551	.869 (.510–1.481)	.607
Rural residence	1.499 (.858–2.622)	.155	1.384 (.848–2.260)	.194
Maternal orphan	.524 (.266–1.030)	.061	.619 (.349–1.095)	.100
Paternal orphan	.773 (.461–1.296)	.329	.722 (.464–1.122)	.147
Lives with biological caregiver	.726 (.370–1.426)	.353	.803 (.454–1.422)	.452
Knows own HIV-positive status	1.396 (.640–3.049)	.402	1.236 (.772–1.979)	.377
Mode of infection—horizontal	1.076 (.618–1.872)	.796	.958 (.501–1.830)	.896
Pregnancy	5.717 (2.507–13.033)	≤.001	Not entered in model	
STI symptomology	Not entered in model		1.542 (1.000–2.380)	.050

### Associations of Individual Social Protection Provisions with Unprotected Sex (Table 4)

Table 4 shows the results of the multivariate regression model of the included social protection provisions. In the full sample, ‘cash-in-kind’ provision of school access (OR 0.52 95 % CI 0.33–0.82 *p* = 0.005), ‘care’ good parental supervision (OR 0.54 95 % CI 0.33–0.90 *p* = 0.019), and adolescent-sensitive ‘care’ at the clinic (OR 0.43 95 % CI 0.25–0.73 *p* = 0.002) were associated with less unprotected sex.

**Table 4:** Logistic regression of all social protection provisions and covariates

Factors	OR (95 % CI)	p
Outcome: unprotected sex (full sample of HIV-positive adolescents n = 1060)		
Age	1.644 (1.476–1.830)	≤.001
Gender	5.727 (3.339–9.824)	≤.001
Informal housing	.927 (.532–1.614)	.788
Rural residence	1.447 (.865–2.422)	.159
Maternal orphan	.596 (.331–1.074)	.085
Paternal orphan	.711 (.451–1.121)	.142
Lives with biological caregiver	.737 (.408–1.332)	.312
Knows own HIV-positive status	.956 (.476–1.921)	.900
Mode of infection—horizontal	1.272 (.778–2.079)	.337
Cash-in-kind—past-week food security	.778 (.459–1.318)	.351
Cash-in-kind—access to school	.523 (.333–.823)	.005
Cash-in-kind—clothing	1.051 (.638–1.733)	.844
Care—positive parenting	1.471 (.936–2.314)	.095
Care—parental supervision	.544 (.327–.904)	.019
Care—HIV support group	1.472 (.828–2.616)	.188
Care—adolescent-sensitive clinic care	.429 (.254–.726)	.002

### Gender Effects (Tables 5, 6)

**Fig. 1:**
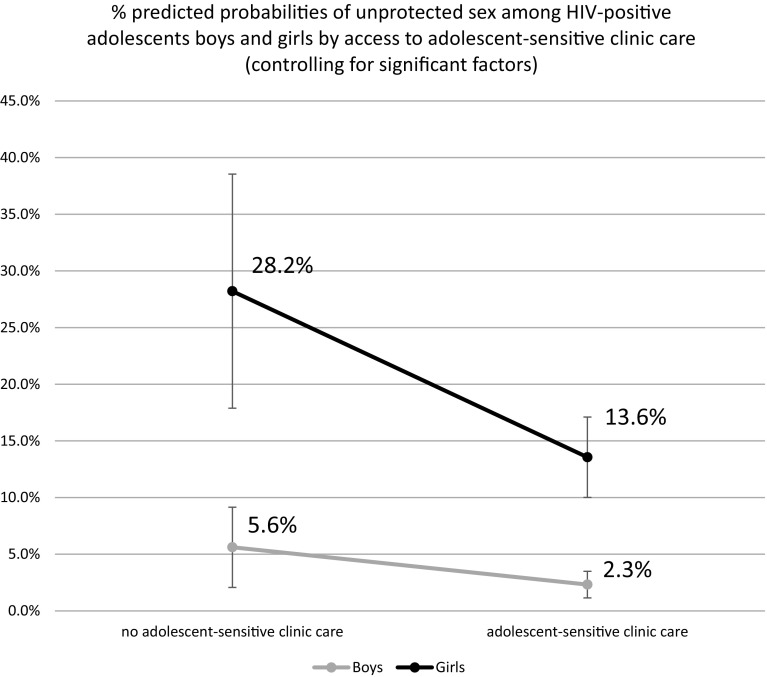
Effects of adolescent-sensitive clinic care by gender

Of all social protection provisions only the interaction between gender and adolescent-sensitive clinic care was significant (OR 0.08 95 % CI 0.01–0.69 *p* = 0.021), suggesting that the effect of adolescent-sensitive clinic care on reducing unprotected sex was significantly greater among HIV-positive adolescent girls than boys (Fig. 1): adjusted probabilities of reporting unprotected sex among HIV-positive girls who accessed adolescent-sensitive clinic services was 14 % compared to 28 % among those who did not. The effect of accessing adolescent-sensitive clinic services was weaker among HIV-positive adolescent boys: with access to services 3 % were likely to report unprotected sex compared to 6 % among those who did not report adolescent-sensitive clinic services.

**Table 5:** Gender moderation effects for HIV-positive adolescents (n = 1060)

Social protection provisions	Outcome: unprotected sex^a^							
	Age		Gender		Social protection		Gender × social protection^a^	
	OR (95 % CI)	p	OR (95 % CI)	p	OR (95 % CI)	p	OR (95 % CI)	p
Past-week food security	1.734 (1.591–1.890)	≤.001	8.045 (2.788–23.213)	≤.001	.907 (.093–8.842)	.933	.832 (.247–2.801)	.766
Access to school	1.713 (1.571–1.867)	≤.001	8.031 (3.581–18.010)	≤.001	.852 (.121–6.013)	.872	.771 (.269–2.209)	.628
Clothing	1.747 (1.602–1.905)	≤.001	10.012 (4.246–23.608)	≤.001	1.886 (.258–13.793)	.532	.608 (.208–1.781)	.365
Positive parenting	1.753 (1.607–1.912)	≤.001	6.492 (3.322–12.688)	≤.001	.701 (.099–4.994)	.723	1.260 (.438–3.622)	.668
Parental supervision	1.718 (1.574–1.874)	≤.001	7.390 (4.009–13.622)	≤.001	.783 (.096–6.395)	.820	.809 (.259–2.531)	.716
HIV support group	1.749 (1.605–1.907)	≤.001	7.777 (4.326–13.981)	≤.001	2.034 (.210–19.673)	.540	.765 (.220–2.667)	.675
Adolescent-sensitive clinic care	1.703 (1.562–1.858)	≤.001	62.987 (7.708–514.724)	≤.001	46.297 (.679–3157.684)	.075	.078 (.009–.685)	.021

^a^ Results for logistic regression models including age, gender, social protection provision and the interaction term for gender and each social protection term

**Table 6:** Gender-disaggregated logistic regressions of social protection provisions and covariates

Factors	Unprotected sex (HIV-positive adolescent girls)		Unprotected sex (HIV-positive adolescent boys)	
	OR (95 % CI)	p	OR (95 % CI)	p
Age	1.667 (1.474–1.887)	≤.001	1.559 (1.228–1.979)	≤.001
Informal housing	.878 (.474–1.626)	.679	1.168 (.287–4.759)	.828
Rural residence	1.537 (.854–2.766)	.152	1.030 (.291–3.646)	.964
Maternal orphan	.450 (.221–.917)	.028	1.483 (.446–4.932)	.520
Paternal orphan	.761 (.451–1.285)	.307	.582 (.204–1.660)	.312
Caregiving arrangement	.654 (.321–1.330)	.241	.958 (.269–3.407)	.947
Mode of infection—horizontal	1.402 (.793–2.479)	.245	1.033 (.360–2.967)	.952
Knows own HIV-positive status	1.152 (.516–2.571)	.729	.627 (.142–2.770)	.538
Cash-in-kind—past-week food security	.868 (.474–1.590)	.648	.629 (.185–2.137)	.458
Cash-in-kind—access to school	.489 (.290–.823)	.007	.638 (.228–1.789)	.393
Cash-in-kind—clothing	.958 (.535–1.717)	.886	1.195 (.398–3.582)	.751
Care—parental supervision	.542 (.300–.982)	.043	.606 (.207–1.778)	.362
Care—positive parenting	1.616 (.958–2.725)	.072	1.019 (.370–2.809)	.971
Care—HIV support group	1.512 (.764–2.992)	.236	1.622 (.521–5.049)	.404
Care—adolescent-sensitive clinic care	.317 (.174–.579)	≤.001	3.598 (.428–30.229)	.238

In subsequent gender-disaggregated regression analyses (Table 6), lower odds of unprotected sex among HIV-positive girls were significantly associated with three social protection provisions: school access (OR 0.49 95 % CI 0.29–0.82 *p* = 0.007), good parental supervision (OR 0.54 95 % CI 0.30–0.98 *p* = 0.043) and adolescent-sensitive clinic care (OR 0.32 95 % CI 0.17–0.58 *p* ≤ 0.001). No social protection provisions were associated with unprotected sex amongst HIV-positive boys.

### Potential Interactive and Additive Effects (Tables 7, 8)

No significant interactive/multiplicative effects of social protection provisions were found in the full sample or for adolescent girls.

**Table 7:** Logistic regression models of all significant potential social protection factors, interaction terms, and covariates

Outcome: unprotected sex	All HIV-positive adolescents (n = 1060)			
Step 1	OR (95 % CI)	p	∆R^2^	p
Age	1.650 (1.512–1.801)	≤.001	.517***	≤.001
Gender	6.226 (3.683–10.523)	≤.001		
Maternal orphan	Not included			
Cash-in-kind—school access	.530 (.349–.804)	.003		
Care—good parental supervision	.616 (.383–.992)	.046		
Care—adolescent-sensitive clinic care	.424 (.254–.707)	.001		

Step 2	OR (95 % CI)	p	∆R^2^	p
Age	1.653 (1.514–1.806)	≤.001	.517	.906
Gender	6.329 (3.729–10.741)	≤.001		
Maternal orphan	Not included			
Cash-in-kind –school access	.457 (.183–1.142)	.094		
Care—good parental supervision	1.831 (.477–7.031)	.378		
Care—adolescent-sensitive clinic care	.435 (.195–.970)	.042		
Interaction—school access × parental supervision	.740 (.281–1.951)	.543		
Interaction—school access × adolescent-sensitive clinic care	1.359 (.486–3.798)	.558		
Interaction—parental supervision × adolescent-sensitive clinic care	.336 (.087–1.297)	.114		

Step 3	OR (95 % CI)	p	∆R^2^	p
Age	1.653 (1.513–1.806)	≤.001	.516	.926
Gender	6.332 (3.731–10.747)	≤.001		
Maternal orphan	Not included			
Cash-in-kind –school access	.447 (.167–1.195)	.109		
Care—good parental supervision	1.703 (.281–10.327)	.563		
Care—adolescent-sensitive clinic care	.428 (.184–.996)	.049		
Interaction—school access × parental supervision	.850 (.071–10.155)	.898		
Interaction—school access × adolescent-sensitive clinic care	1.398 (.452–4.323)	.561		
Interaction—parental supervision × adolescent-sensitive clinic care	.366 (.052–2.603)	.316		
Interaction—school access × parental supervision × adolescent-sensitive clinic care	.849 (.057–12.578)	.905

**Table 8:** Logistic regression models of all significant potential social protection factors, interaction terms, and covariates

Outcome: unprotected sex	HIV-positive adolescents girls (n = 584)			
Step 1	OR (95 % CI)	p	∆R^2^	p
Age	1.699 (1.536–1.880)	≤.001	.528***	≤.001
Gender	Not included			
Maternal orphan	.587 (.361–.955)	.032		
Cash-in-kind—school access	.515 (.318–.833)	.007		
Care—good parental supervision	.634 (.364–1.103)	.107		
Care—adolescent-sensitive clinic care	.313 (.174–.564)	≤.001		

Step 2	OR (95 % CI)	p	∆R	p
Age	1.716 (1.547–1.903)	≤.001	.537	.146
Gender	Not included			
Maternal orphan	.591 (.362–.966)	.036		
Cash-in-kind –school access	.513 (.182–1.443)	.206		
Care—good parental supervision	3.634 (.714–18.490)	.120		
Care—adolescent-sensitive clinic care	.392 (.160–.964)	.041		
Interaction—school access × parental supervision	.738 (.235–2.321)	.604		
Interaction—school access × adolescent-sensitive clinic care	1.147 (.354–3.720)	.819		
Interaction—parental supervision × adolescent-sensitive clinic care	.158 (.030–.819)	.028		

Step 3	OR (95 % CI)	p	∆R^2^	p
Age	1.714 (1.545–1.902)	≤.001	.537	.785
Gender	Not included			
Maternal orphan	.588 (.359–.962)	.035		
Cash-in-kind—school access	.487 (.162–1.464)	.200		
Care—good parental supervision	3.006 (.361–25.029)	.309		
Care—adolescent-sensitive clinic care	.378 (.147–.969)	.043		
Interaction—school access × parental supervision	1.092 (.053–22.650)	.955		
Interaction—school access × adolescent-sensitive clinic care	1.229 (.343–4.407)	.752		
Interaction—parental supervision × adolescent-sensitive clinic care	.197 (.020–1.950)	.165		
Interaction—school access × parental supervision × adolescent-sensitive clinic care	.634 (.024–16.674)	.785		

**Fig. 2:**
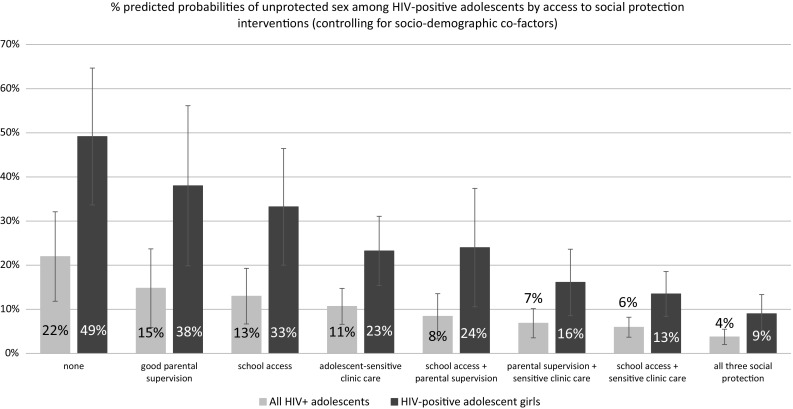
Marginal effects model testing for additive effects of combination social protections on unprotected sex among HIV-positive adolescents

However, the independently significant effects of social protection provisions in Table 4 suggested potential additive effects. Strong additive effects were shown in the full sample and among HIV-positive adolescent girls. Among all HIV-positive adolescents, who had no access to school, good parental supervision, nor adolescent-sensitive clinic care, 22 % reported unprotected sex at last intercourse. Those receiving one social protection 11–15 % reported unprotected sex, and with any two: 6–8 % probability of unprotected sex. Adolescents receiving all three social protection provisions were likely to report just under 4 % unprotected sex. Amongst HIV-positive girls, rates of unprotected sex dropped from 49 % with no social protection provisions, to 23–38 % with one, 13–24 % with two and just under 9 % with all three social protection provisions (Fig. 2). As no social protection provisions were significantly associated with unprotected sex among HIV-positive boys, marginal effects models were not conducted.

## Discussion

Findings from this study have several important implications. First, we found high rates of unprotected sex reported by HIV-positive adolescents, and significantly higher rates of virological failure amongst HIV-positive adolescents engaging in unprotected sex, suggesting greater transmission risk to uninfected peers. It is clear that effective programming to reduce sexual risk behavior for this vulnerable group is essential.

Second, we identify three types of social protection provisions that are strongly associated with reduced unprotected sex among HIV-positive adolescents: access to schools, good parental supervision, and adolescent-sensitive sexual health care at clinics. These findings reflect emerging evidence on combinations of social protection for reducing sexual risk-taking among general samples of adolescents [23]. They support recent calls for adolescent-sensitive HIV-inclusive social protection, that is social protection that reaches HIV-positive and HIV-affected adolescents without using HIV status as a targeting condition [21]. This study’s results show that HIV-inclusive social protection has the potential to reduce HIV risk-taking without the associated stigma of HIV-specific interventions.

Third, we extend this existing research by showing that combining two types of social protection: ‘cash-in-kind’ (school access) and ‘care’ (good parental supervision and adolescent-sensitive sexual health clinic care) has the greatest potential to reduce unprotected sex the most. Compared to those receiving none or one social protection provision, adolescents who receive two types of social protection reported lower rates of unprotected sex, with those receiving three types of social protection reporting the lowest rates. These findings suggest that ‘care’ social protection may act as the ‘glue’ for cash social protection to have positive effects, or vice versa. Additional research is needed to elucidate these potential mechanisms.

Fourth, our findings highlight the importance of receiving social protection in three key locations for adolescents: school, home and clinic. These findings confirm evidence from the region on adolescents more generally, with access to school serving as a ‘social vaccine’, bolstering social pathways associated with improved resilience [13]. Additionally, receiving adolescent-sensitive ‘care’ services from sexual healthcare providers at clinics was also associated with lower rates of unprotected sex. This finding supports qualitative reports from South Africa on the negative effect of poor clinic care on adolescent sexual and reproductive health outcomes [43]. Further analyses, including in-depth qualitative research, are needed to better understand the mechanisms through which classroom- and clinic-level support is linked to reduced unprotected sex.

Fifth, our gender-disaggregated analyses resulted in different significant social protection for boys and girls, though this may also be due to reduced power and the lower rates of sexual activity reported by the HIV-positive adolescent boys in our sample [15]. Three of the social protection provisions we tested have significant effects on HIV-positive adolescent girls: access to schools, good parental supervision, and adolescent-sensitive sexual health clinic care. Supporting adolescent girls beyond the home setting, at school and clinics, will not only ensure they reach services critical to their long-term well-being, but also support them in engaging in safer sex. Notably, these three provisions are—when available—targeted at all adolescents, whether HIV-positive or not. This suggests that social protection that reaches at-risk populations such as adolescents, even when not targeted to HIV-positive ones, can be effective to reduce their vulnerabilities. These findings resonate with advocacy for generalised social protection in the Sustainable Development Goals [13]. They also underline the importance of ensuring that HIV-positive adolescents are not excluded from accessing social protection.

This study has several methodological limitations. Cross-sectional analyses always limit our ability to reach conclusions on the direction of the observed associations, due to potential reverse causality for significant associations. Future research can valuably test these associations in longitudinal quasi-experimental studies or randomised controlled trials. Second, self-reported sexual health outcomes contain risk of social desirability bias. As a check for validity, we tested associations of self-reported unprotected sex with two other sexual and reproductive health outcomes. Unprotected sex was significantly associated with pregnancy and STI symptomology. Third, although over 90 % of all eligible adolescents in the health district were included in this sample, it is possible that adolescents at highest risk were those who refused or were untraceable. However, comparison of the sample reached and those not reached showed no significant differences by age, gender and residential location—the only information available to us. Despite this limitation, our study is the first and largest study of HIV-positive adolescents traced into their homes and communities, and thus may allow more representativity of the overall population than clinic-based samples that are thus restricted to those who attend healthcare services. Moreover, by including study sites with high HIV prevalence and relatively poor resources, our findings may be applicable to contexts with similar socio-economic and epidemiological profiles.

Participants in our sample reported very high coverage of certain social protection provisions: social cash transfers and school feeding (>90 %). These coverage rates not only limited our ability to conduct sub-group analyses but also precluded us from reaching any conclusions on whether they may be associated with sexual health outcomes among HIV-positive adolescents. However, given prior evidence from South Africa on the effectiveness of social cash transfers in reducing sexual risk-taking among AIDS-affected adolescents [24, 44], our findings suggest that the positive effect of additional social protection may extend gains from the social cash transfer and school feeding schemes documented by prior studies in the region.

Despite the above limitations, the study provides key insights for sexual health programming among HIV-positive adolescents in and out of clinical care. The interventions identified are available in real-life settings and have statistically and practically significant associations with reduced unprotected sex, particularly when accessed in combination. Increasing access to these social protection provisions among HIV-positive adolescents has the potential to support HIV-positive adolescents to reduce unprotected sex, and its related outcomes of unwanted pregnancies and onwards HIV-transmission.
